# Use of rapid diagnostic tests (RDTs) for conclusive diagnosis of chronic Chagas disease – field implementation in the Bolivian Chaco region

**DOI:** 10.1371/journal.pntd.0007877

**Published:** 2019-12-19

**Authors:** Daniel Lozano, Lizeth Rojas, Susana Méndez, Aina Casellas, Sergi Sanz, Lourdes Ortiz, María Jesús Pinazo, Marcelo Abril, Joaquim Gascón, Faustino Torrico, Julio Alonso-Padilla

**Affiliations:** 1 Fundación CEADES, Cochabamba, Bolivia; 2 Universidad Mayor de San Simón, Cochabamba, Bolivia; 3 Barcelona Institute for Global Health (ISGlobal), Hospital Clínic—University of Barcelona, 08036 Barcelona, Spain; 4 Plataforma de Chagas Tarija—Universidad Autónoma Juan Misael Saracho, Tarija, Bolivia; 5 Fundación Mundo Sano, Buenos Aires, Argentina; Universidade Federal de Minas Gerais, BRAZIL

## Abstract

Chagas disease, caused by the parasite *Trypanosoma cruzi*, is the neglected tropical disease with a highest burden in Latin America. Its acute stage is mostly asymptomatic and goes unnoticed. Symptoms appear at the chronic stage, which is when diagnosis is usually made. This is based on the agreement of two conventional serological tests such as Enzyme-Linked Immunosorbent Assays (ELISAs). There are commercial kits with good sensitivity and specificity but their use is impractical in many highly endemic regions with poorly equipped laboratories. Luckily, several rapid diagnostic tests (RDTs) are available for the detection of anti-*T*. *cruzi* immunoglobulins. They are easy to operate, require no cold storage, provide fast turnaround of results, and some can work with a tiny volume of whole blood as sample. With the aim to field validate their use we compared an alternative algorithm based on a combination of RDTs with the standard based on ELISAs. In both cases a third test was available in case of discordance. RDTs were implemented by mobile teams in field campaigns to detect chronic *T*. *cruzi-*infections in the Chaco region of Bolivia. ELISAs were made in the reference laboratories located in the main hospitals of Yacuiba and Villa Montes, two major cities of the region. We enrolled 685 subjects who voluntarily participated in the study and had not been treated against the disease before. The agreement between the two main RDTs was 93.1% (638/685) (kappa index = 0.86; CI 95% 0.83–0.90). In comparison to the ELISAs algorithm, the combined use of the RDTs provided a sensitivity of 97.7% and a specificity of 96.1%. These results support the use of RDTs for the diagnosis of chronic Chagas disease in the studied region, and encourage their evaluation in other regions of Bolivia and other endemic countries.

## Author summary

Chagas disease is caused by the parasite *Trypanosoma cruzi*. It affects ~7 million people worldwide, exerting its highest impact in Latin America. The acute stage of the infection is generally asymptomatic and goes undiagnosed and untreated. It is in the long lasting chronic stage that the life-threatening heart and/or gut tissue disruptions may occur. Then diagnosis is performed by serological detection of *T*. *cruzi-*specific immunoglobulins in the patients´ sera, usually upon suspicion of the clinical symptomatology. Such diagnosis may arrive too late for those with advanced tissue damage as there are evidences indicating that the treatment might be ineffective for them. It is thus of huge importance to provide an early diagnosis to get access to treatment. But the use of current conventional serological tools is not possible in many highly endemic regions distant from equipped reference laboratories. Herein we evaluate an alternative algorithm for the conclusive diagnosis of chronic Chagas disease based on the use of rapid diagnostic tests (RDTs) in a field study made in the Chaco region of Bolivia. Comparison of the RDTs performance with that of the ELISAs “gold-standard” yielded a sensitivity of 97.7% and a specificity of 96.1%. These values justify a wider use of the RDTs in the region under study, and encourage their evaluation in other regions of Bolivia and other endemic countries.

## Introduction

Chagas disease, caused by infection with the protozoan parasite *Trypanosoma cruzi* (*T*. *cruzi*), is the neglected tropical disease exerting a highest burden in the Western hemisphere [1]. It is estimated that there are ~7 million people affected worldwide, the majority of them living in Latin America where the insect vectors that transmit the infection are endemic [1]. Besides, vector-independent transmission routes like blood-transfusion, organ transplant, and, very importantly, from mother-to-child have also been described [2]. These are of relevance in endemic and non-endemic countries (like Spain), where the disease impact has spread to in the last decades due to population movements [3].

Three clinical stages are distinguished in the progression of Chagas disease. First there is a short lasting acute phase that is mostly asymptomatic and goes unnoticed. It is followed by a long lasting chronic phase that may span for decades without showing any symptoms associated to the infection (indeterminate stage). About 40% of those chronically infected will end up developing life-threatening disruptions to the heart and/or digestive tract tissues (determinate stage); lesions that can lead to death if left untreated [2].

Chagas suspicion can be made by observation of its associated symptomatology, but serological confirmation following WHO/PAHO criteria is mandatory in order to establish a diagnosis [4, 5]. However, it is fundamental to achieve it before this is advanced as the anti-parasitic treatments available (benznidazole and nifurtimox), efficacious against the parasite, cannot revert the damage to the tissues [6]. Both drugs have a very good efficacy against the acute stage of the infection and are well tolerated by children [1, 2]. Unfortunately their efficacy diminishes in the chronic stage of the infection and the advent of adverse events linked to their long administration regimes is frequent in adults [7, 8]. Nevertheless, they are the only drugs available at present, and several studies have proved the benefits of their administration [6, 9–12]. But, despite the advantages of treatment, it is estimated that less than 1% of those infected eventually get access to it [13], due to a series of factors amongst which overarches the lack of a timely diagnosis [14]. Thereby, propelling a drive to this dramatic figure should be immediately addressed. A goal further supported by the acknowledged benefits of drug administration to women at childbearing age towards the prevention of congenital transmission [14].

Methodologies used to diagnose *T*. *cruzi* infection largely depend on the disease stage status. In the acute phase, the levels of parasitemia enable direct detection of the parasite either by parasitological or by molecular biology techniques [15]. Parasitemia becomes low and intermittent in the chronic phase, but the acute infection triggered seroconversion and anti-*T*. *cruzi* specific immunoglobulins are detectable for years, so the chronic stage can be indirectly identified by serological methods. The most commonly used is the enzyme-linked immunosorbent assay (ELISA), of which there are many kits commercially available [16]. Their sensitivity and specificity are generally very good, but they work with serum or plasma samples which involve blood extraction by venous puncture and a centrifugation step to segregate sera or plasma from other blood components. Besides, plasma and/or serum samples require of cold storage, similarly to some of the components of the ELISA kits. Furthermore, ELISAs must be performed by technically trained personnel and their results preferentially read with a spectrophotometer. All these features are common to other conventional serological methods for Chagas disease diagnosis like indirect immunofluorescence (IIF). Unfortunately such conditions are often unattainable in vast areas highly endemic for the disease that only count with poorly equipped laboratories [14]. Moreover, under the recently confirmed WHO/PAHO recommendation on the agreement of two techniques with distinct antigen sets for conclusive *T*. *cruzi* diagnosis [4, 5], results turnaround of conventional diagnostics may delay for several weeks due to logistical and operational matters. Plus it usually involves more than one visit to the hospital, which is unfeasible for a population with low-resources that often live far away from it. As a consequence, many people who could be targeted for treatment remain undiagnosed, and untreated.

Fortunately there are several rapid diagnostic tests (RDTs) commercially available for the diagnosis of chronic Chagas disease [17]. These were developed to be used as point-of-care (PoC) diagnostics in the context of highly endemic settings that lack of the appropriate laboratory resources [18]. Various studies have previously described they can provide very good sensitivity and specificity, even working with tiny volumes of whole blood [19–22]. Furthermore, the combined use of two RDTs has already been suggested as a substitute of the current algorithm based on conventional serological methods [19, 23]. In the present study we demonstrate that the field use of RDTs by mobile teams could indeed substitute the use of conventional tests in the highly endemic Chaco region of Bolivia.

## Methods

### Study design, location, and sampling of participants

In order to compare the performance of RDTs with that of ELISAs, the statistical assumption was that the former´s was at least as good as the latter´s. For an expected kappa index (κ) of 0.99 (± 0.01) between both methods, an error margin of the estimation of κ of 1% and an expected prevalence of the event (*T*. *cruzi* infection positive) of 6% [24], the necessary sample size was of 680 subjects [25].

Enrollment was prospectively offered to all patients >1 year old that had not been previously treated for *T*. *cruzi* infection. It generally took place in the context of field screening campaigns performed in communities around the cities of Yacuiba and Villa Montes (province of Gran Chaco, department of Tarija). These campaigns were previously arranged with the community leaders and adequately advertised. Sampling was made between April 2018 and August 2018 and the number of people that could be screened by the field teams in a single day ranged from 18 to 60 depending on the campaign. There were two field teams for the RDTs campaigns, one working in the area of Yacuiba and the other in the area of Villa Montes. These included an operator, responsible for the performance of the RDTs and their interpretation, and a support team to help out with the activities of information, education and communication (IEC) of the study and participants enrolment.

All subjects were seen in a single visit that entailed: (1) communication, explanation and information of the study and signature of its informed consent; (2) performance of the RDTs from finger pricked whole blood samples was made in terrain by trained personnel and the data and results obtained with RDTs were registered in situ with electronic tablets (ODK Collect software; version v1.22.4) [26]; and (3) extraction of a larger volume of blood (~3 ml) by venous puncture to be taken to the laboratory for isolation of serum. The impact of features such as hemolysis was negligible. Serum samples were used immediately to perform the ELISA tests in the hospital laboratories or stored frozen until needed. Study participants were appointed for a results pick up visit in the forthcoming weeks upon sampling to communicate them the outcome of the ELISAs algorithm.

### Ethics

The study complied with the principles of the Declaration of Helsinki. It was presented for its acknowledgment and approval to the Departmental Chagas disease Program and to the Universidad Autónoma Juan Misael Saracho, which are respectively the Ministry of Health authority and the main academic institution in Tarija (Bolivia). Besides, the study protocol was reviewed and approved by the Ethical Committees of Fundación CEADES (Cochabamba, Bolivia) and Hospital Clínic of Barcelona (Barcelona, Spain). All patients included in the study signed an informed consent. For those under 18 years old, the inform consent was signed by the mother, father or assigned tutor. All the participants who were diagnosed as positive by ELISA were directed to the corresponding medical doctor to be considered for treatment accordingly to medical criteria.

### ELISAs and RDTs

We used the same Wiener recombinant and Wiener lysate antigen ELISA tests as described before [19]. In case of discordance between them, a third ELISA test was used (Chagatek, Laboratorio Lemos, Buenos Aires, Argentina). All three tests were performed with serum samples following the manufacturers´ instructions. Sera were obtained by centrifugation of coagulated whole blood obtained by venous puncture.

We also used the same RDTs as described before [19]: Chagas Stat-Pak (CSP; Chembio Inc., Medford, USA) and Chagas Detect Plus (CDP; InBIOS International Inc., Seattle, USA). In case of discordance between them, WL-Check RDT kit (Wiener Laboratorios, Buenos Aires, Argentina) was used [27]. All three RDTs were performed simultaneously with a tiny volume of whole blood obtained by finger prick and interpreted following the instructions provided by the manufacturers.

### Data collection, study variables and analysis

Study participants´ visit data and RDTs qualitative outcome were immediately collected with a questionnaire devised for the study in the ODK Collect software installed in two electronic tablets, one per mobile team. Batch laboratory results from the ELISA tests were introduced in the OpenClinica platform (v3.13) for their safe storage and subsequent analysis [28]. This consisted in crossing the ODK and OpenClinica datasets and determining the performance of RDTs in comparison to the conventional serological tests, the ELISAs, which were taken as “gold-standard”. The following statistical measures were considered: (i) sensitivity, correctly classified positive subjects; (ii) specificity, correctly classified negative subjects; (iii) positive predictive value (PPV), the proportion of positive patients that were really positive; (iv) negative predictive value (NPV), the proportion of negative patients that were really negative; and (v) diagnostic efficiency (DE), the proportion of individuals correctly classified.

Data were described as frequencies and mean (with standard deviation, SD) for discrete and continuous variables, respectively. The agreement between classification tools was estimated using the kappa statistic (κ) [29]. The κ measure of agreement is scaled to be 0 when the amount of agreement is what would be expected to be observed by chance and 1 when there is perfect agreement [30]. Specifically, κ <0, no agreement; κ = 0–0.20, poor agreement; κ = 0.21–0.40, fair agreement; κ = 0.41–0.60, moderate agreement; κ = 0.61–0.80, substantial agreement; and κ = 0.81–1.00, nearly perfect agreement. The significance level was set at 0.05, and the analysis was carried out using Stata [31].

## Results

### Description of the study population

**Fig 1:**
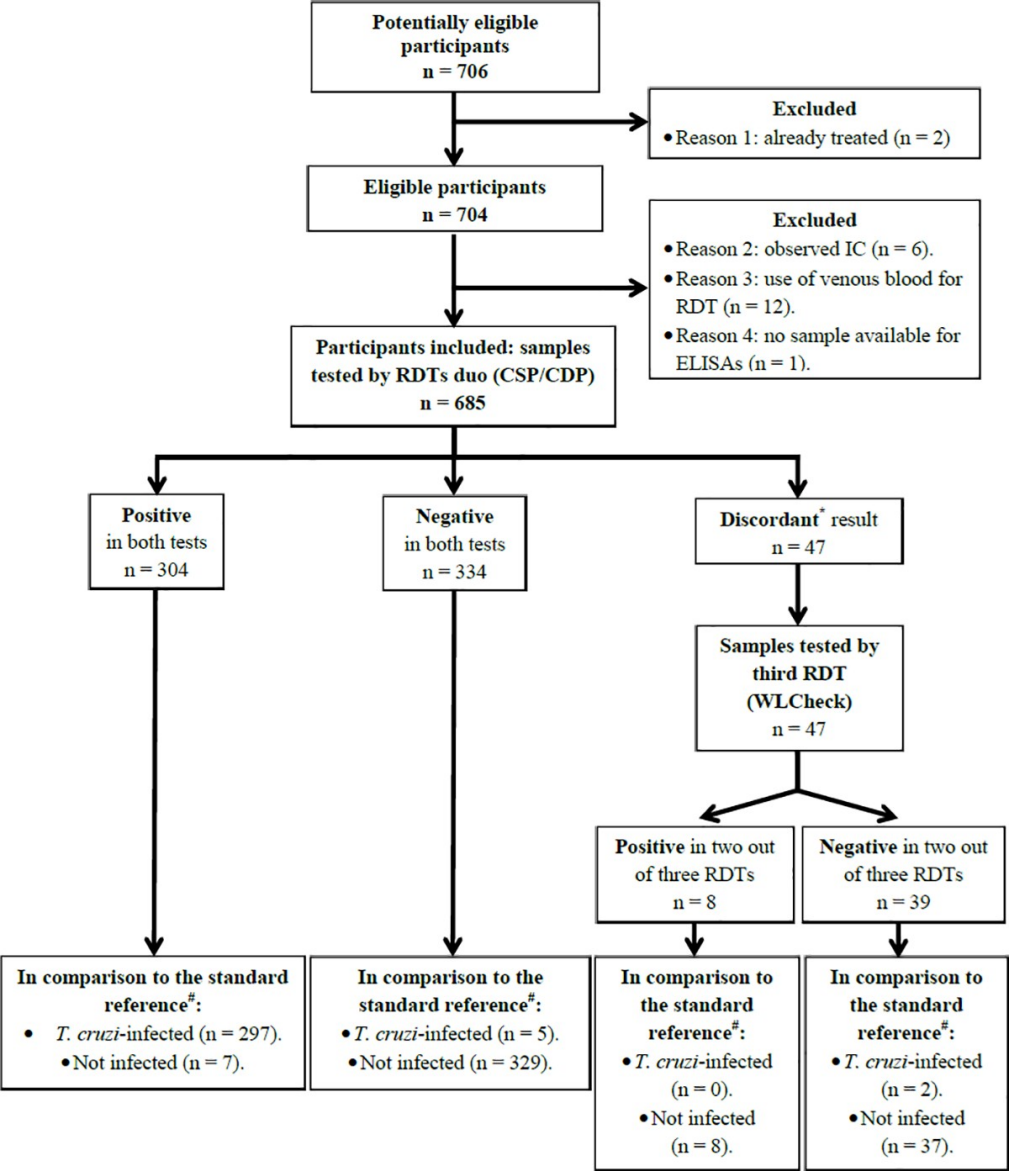
Study flowchart depicting the excluded subjects and the RDTs and ELISAs results of all of those included. *Amongst the 47 discordant samples between the two main RDTs: 3 were CSP positive and CDP negative, and 44 were CSP negative and CDP positive; rate of disagreement = 6.9%. Hence, following the recommended algorithm, a third RDT based on distinct antigen set was used and the agreement of two out of three techniques considered. ^#^The standard reference is the algorithm based on the agreement of two ELISAs, as recommended by the WHO/PAHO [4, 5].

Study sampling and testing were made between April 2018 and August 2018. Upon applying inclusion and exclusion criteria, 685 subjects were finally included (Fig 1). In relation to the origin of the subjects, 68.2% (467/685) had been born in the department of Tarija and the remaining 31.8% originated from other departments of Bolivia (218/685), being the most represented those from Chuquisaca (120/218) and Santa Cruz (41/218) departments.

Mean age of participants was 31.0 years old (SD = 19.4). In total, 75% of the samples were from subjects *≥*15 years old (514/685; Table 1). The rest comprised 53 samples from subjects under 5 years old (7.7%) and 118 from subjects within the range of 5 to 14 years old (17.3%). In terms of gender, 242 were male and 443 female (35.3% and 64.7%, respectively; Table 1). None of the subjects included in the study had received anti-parasitic treatment against *T*. *cruzi* infection.

**Table 1:** Brief description of the population participating in the study.

Average Age (years)^1^	31.0 (±19.4) [685]		
Age groups (years)^2^		[1–4]	53 (8%)
		[5–14]	118 (17%)
		[≥15]	514 (75%)
Gender^2^		Male	242 (35%)
		Female	443 (65%)

^1^Arithmetic mean (SD) [N];

^2^N (column percentage).

### Description of the study campaigns

Sampling and RDTs were mostly performed in form of campaigns that took place in communities and municipalities around Villa Montes and Yacuiba. In total, 14 campaigns were performed in and around each city (S1 Table). A total of 12 localizations were campaigned in or around Villa Montes, comprising 8 rural, 3 urban and 1 peri-urban locations (S1 Table). While a total of 8 localizations were campaigned in or around Yacuiba, comprising 6 rural and 2 urban locations (S1 Table).

### Performance of the ELISA tests

When opposing the performance of the two main ELISA tests, Wiener´s based on recombinant and lysate antigens, their percentage of agreement was 96.1% (κ = 0.92; CI 95% 0.89–0.95) (Table 2).

**Table 2:** Performance of the algorithms based on ELISAs or RDTs for the diagnosis of chronic Chagas disease.

Tests	Agreement between tests (%)	Kappa (CI95%)	Discordant results	Seropositive by final algorithm*	Seronegative by final algorithm*
Wiener ELISAs	96.1%	0.92 (0.89–0.95)	27 (3.9%)	304 (44.4%)	381 (55.6%)
CSP *vs* CDP	93.1%	0.86 (0.83–0.90)	47 (6.9%)	312 (45.5%)	373 (54.5%)

*Final algorithm result obtained upon testing discordant samples with the corresponding third test in each case.

There were 27 samples with discordant results that were subjected to a third ELISA (Chagatek, Laboratorios Lemos), which yielded 9 positive and 18 negative determinations. With the support of the third ELISA and taking into account the recommendation on the agreement of two tests for conclusive Chagas disease diagnosis, out of the 685 study participants 304 subjects were described as positive (44.4%), and 381 were determined as negative (55.6%) (Table 2). No significant differences were observed between the results obtained with samples from the sites of Villa Montes in relation to those from Yacuiba.

**Fig 2:**
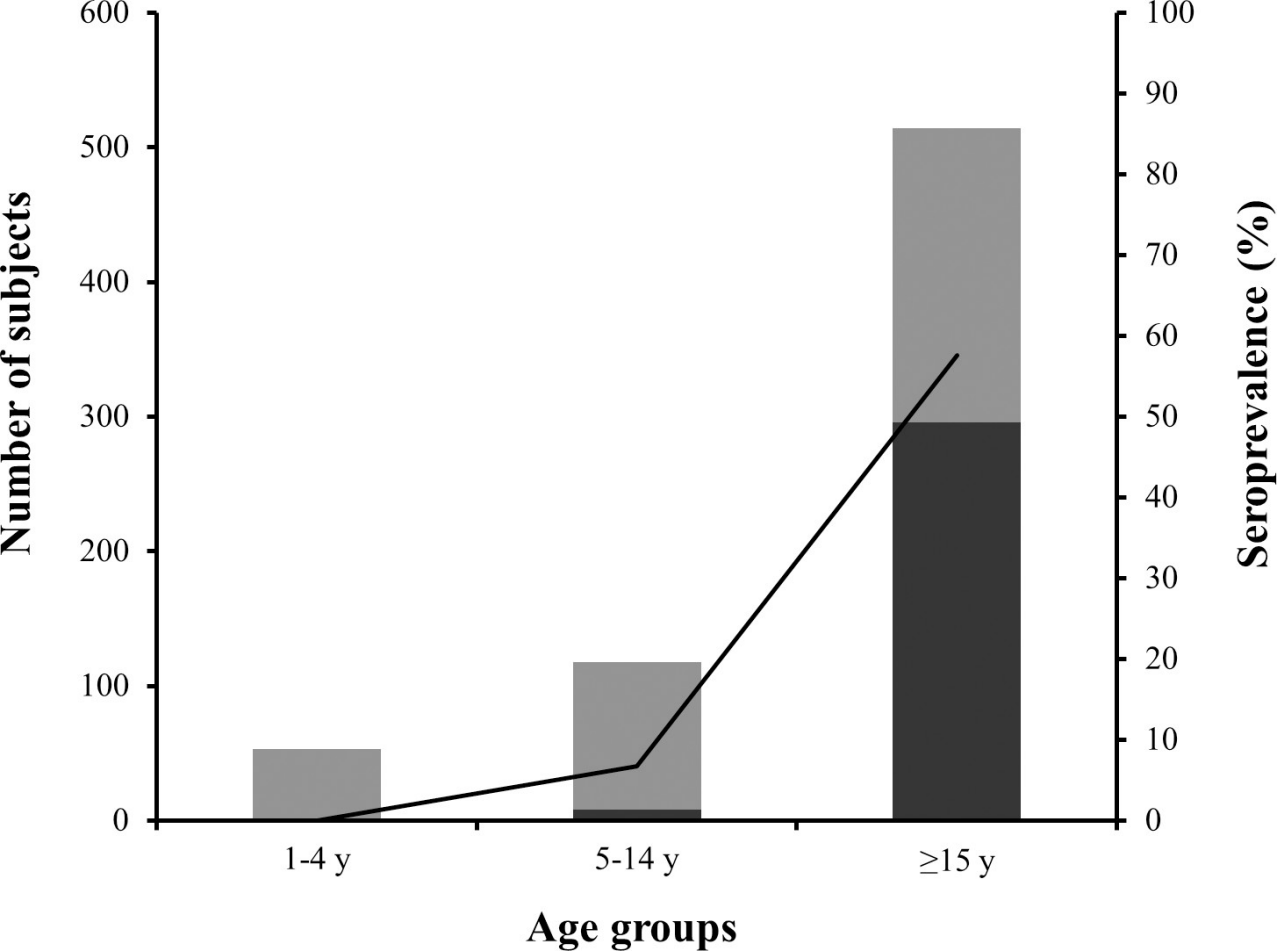
Seroprevalence per age group. Serological status of patients per age group studied. Dark bars show the number of positive samples out of the total samples (whole bars) per age group (scale at left Y-axis); line indicates the % of positive patients per age group (scale at right Y-axis).

Overall, prevalence of Chagas disease in the population under study was determined to be 44.4%. But, as it could be expected, seroprevalence rates varied per age group being lower in the groups of subjects from 1–4 years old (0/53) and from 5–14 years old (8/118; 6.8%), in comparison to the group of subjects ≥ 15 years old (296/514; 57.6%) (Fig 2).

### Performance of the RDTs

When comparing head-to-head the level of agreement between the two main RDTs (CSP and CDP), it appeared that both coincided in 93.1% of their determinations (κ = 0.86; CI95% 0.83–0.90) (Table 2). The use of the third RDT (WL-Check) helped to resolve 47 discordant results: 39 of them were determined to be negative and 8 were positive (Table 2; Fig 1). Again, no significant differences were observed between the results obtained with samples from the sites of Villa Montes in relation to those from Yacuiba.

In order to find which of the two main RDTs had a better performance, we individually compared them with the “gold-standard”, i.e. with the results obtained with the ELISAs algorithm. CSP results agreement to those yielded by the ELISAs was 97.5% with an excellent kappa value (κ = 0.95; CI95% 0.93–0.97) (Table 3). On the other hand the level of agreement of CDP results to the ELISAs output was 92.1% with a very good kappa value (κ = 0.84; CI95% 0.80–0.88) (Table 3).

**Table 3:** Table comparing the results of Chagas Stat Pack (CSP) and Chagas Detect Plus (CDP) individually or taking into consideration their combined use (RDTs) versus the "gold-standard" based on ELISAs.

	ELISAs*	CSP	CDP	RDTs^#^
**False positive**	0	10	49	15
**True positive**	304	297	299	297
**False negative**	0	7	5	7
**True negative**	381	371	332	366
**Total**	685	685	685	685
**% Agreement**	-	97.5	92.1	96.8
**Kappa (CI95%)**	-	0.95 (0.93–0.97)	0.84 (0.80–0.88)	0.94 (0.91–0.96)
**Sensitivity**	-	97.7	98.4	97.7
**Specificity**	-	97.4	87.1	96.1
**PPV**	-	96.7	85.9	95.2
**NPV**	-	98.1	98.5	98.1
**DE**	-	97.5	92.1	96.8

Note that CSP, CDP and RDTs results of “% of agreement” (and kappa value), “sensitivity”, “specificity”, “PPV”, “NPV” and “DE” shown in the table correspond to their comparison to the outcome of using the ELISAs.

*, results obtained with the standard algorithm based on the agreement of two conventional serological tests (ELISAs in this case);

^#^, results obtained with the combined use of up to three RDTs, as described.

In terms of their performance parameters, although the sensitivity of CDP was slightly higher than that of CSP, the latter generally yielded better results than CDP (Table 3). The specificity, PPV and DE provided by CDP in particular were lower than those achieved with CSP, due to the higher number of false positive cases reported with the former.

In case of discordant results between the two main RDTs a third rapid test (WL-Check, Wiener) was used, and the agreement of two of them was considered for conclusive diagnosis as recommended [4, 5]. When we compared the RDTs algorithm (up to three tests) with the “gold-standard” based on the combination of ELISAs (up to three), the use of RDTs yielded a sensitivity of 97.7% (297/304), a specificity of 96.1% (366/381), its PPV and NPV were respectively 95.2% (297/312) and 98.1% (366/373), and it showed a capacity of correctly classifying positive and negative subjects (DE) of 96.8% (663/685) (Table 3).

## Discussion

This work represents the next step of a previous study that described the combined use of RDTs as an alternative to the use of conventional serological methods for the diagnosis of chronic Chagas disease [19]. We aim at validating the implementation of rapid easy-to-use tests so that these can substitute conventional serology, an advancement that would be especially relevant towards the disease diagnosis in highly endemic regions with poorly equipped laboratories. In contrast to the study by Egüez et al., we have now taken the RDTs outdoors and used them in the form of field screening campaigns by mobile teams.

Similarly to what it was reported in the study in Sucre, the seroprevalence reported by this work in the Chaco region around the cities of Yacuiba and Villa Montes was higher than the average Chagas disease seroprevalence reported in Bolivia [24]. This may be due to the fact that the surveyed regions are highly endemic for the disease. Moreover, the offer of a free of charge *T*. *cruzi* infection diagnosis to the population could have had a call-in effect to those people in suspicion of having acquired the disease. In any case, the high seroprevalence rate observed (44.4% of the serum samples analyzed) was very similar to that reported before in studies performed with CSP and ELISAs in the close municipality of Carapari (Gran Chaco province) with people of all ages [22], as well as the rates reported with samples from pregnant adult women by Shah et al. in their field study with CDP and ELISAs in the municipality of Camiri (Cordillera province of the Bolivian Chaco) [21].

In our study, the agreement between the two main RDTs used was high (93.1%), although not as high as the agreement between the two main ELISAs (96.1%). The level of agreement of the RDTs was neither as good as what we had described before for the same kits in the Sucre study (100%) [19]. The lower agreement rate observed now might be due to eco-epidemiological differences of the surveyed areas of the Chaco region in comparison to Sucre peri-urban area, as well as to the fact that the rapid tests were performed outside the lab in a non-controlled environment. It addition, it must be indicated that due to the operational characteristics of the field campaigns, RDTs results were interpreted by a single operator. This may have biased the agreement of the tests due to a lack of independence.

We would like to highlight that we decided to use three different rapid assays, based on distinct antigen sets, in order be able to head-to-head compare their performance to that of the ELISAs algorithm (the “gold-standard”). In fact, the use of a third RDT (WL-Check, Wiener) was needed to untie 47 discordant results. We are aware that an algorithm relying on three RDTs might arguably not be a good option in the terrain due to its cost. Notwithstanding, we also individually compared the performance of each of the two main RDTs with the outcome of the ELISAs algorithm. Remarkably, both RDTs did independently provide a very good performance (see Table 3). CDP provided the highest sensitivity of the two (98.7%, versus 97.7% of CSP); whereas CSP had a very high specificity (97.4%). Notably, CSP yielded a DE of 97.5%, which means that it was capable of correctly classifying almost 98 of each 100 tested subjects. These figures would support the use of CSP by itself as a conclusive diagnosis of chronic *T*. *cruzi* infection. However, following current recommendation, a confirmation of the positively screened cases would be required with another test based on another antigenic set [4, 5]. The use of a conventional test to confirm a positive RDT outcome is already in place in Bolivia [32], and it was recently suggested in a meta-analysis article that reviewed the performance of several RDTs [33]. But we must emphasize that the necessity of a conventional assay to confirm a result obtained with an easy-to-use RDT would leave us in the same position where we are now. Such conventional confirmatory test will have to be made in an equipped laboratory by trained personnel, features that are often unavailable in highly endemic areas. Besides, the person would probably have to wait for several weeks to get a results turnaround with the consequent risk of being loss to follow-up. Thereby, confirmation of a seropositive output with another RDT would be much more desirable in this context. Even more, taking into consideration the very good performance obtained using just one diagnostic test in a highly endemic region like the one studied here, it would make full sense to adapt the current chronic Chagas disease diagnosis policy to the regional epidemiological reality.

In light of the results obtained by Egüez et al. [19] and in this study with any of the two main RDTs using a small volume of whole blood as sample, the use of a RDT could be applied as secondary confirmative tool, similarly to what is already done for the diagnosis of HIV infections [34]. The referenced document even contemplates “[…] the potential of HIV self-testing to increase access to and coverage of HIV testing […]” [34]. Such procedure could also be considered for Chagas disease, particularly when using CSP (Chembio Inc., Medford, USA) because it self-contains the required elements to conduct the diagnosis (like a blood-drop dispenser to add the sample into the diagnostic cassette). Operational robustness and easy-to-use are very important features to consider when working in the field. Unfortunately, this would not be the case for CDP (InBIOS Inc, Seattle, USA) as it is now, since it lacked the dispenser and was operationally more complicated to use.

Although we have obtained very good results using RDTs in Sucre [19] and in the Bolivian Chaco (this study), RDTs have been previously described to have limitations to their performance depending on the geographic region where they are used [35]. For instance a poorer performance in regions with lower *T*. *cruzi* prevalence (Arequipa area in Peru versus Santa Cruz area in Bolivia) has been described [35]. This would be explained by a potential direct relationship between RDTs sensitivity and the levels of anti-*T*. *cruzi* immunoglobulins in the studied population [35]. It could as well be due to the genetic background of the predominant parasite strains: the RDTs performing better in those regions with circulating parasites antigenically closer to the strains mirrored to design the kits [35]. Other feature that might influence the RDTs performance could be the occurrence of cross-reactivity events when evaluating them with samples from *Leishmania* spp. or *T*. *rangeli* infected subjects. This is particularly relevant in case RDTs had to be used in regions where these parasites co-exist with *T*. *cruzi*. To our knowledge, studies dealing with this potential issue have not been published yet, although the opposite, cross-reactivity of a Chagas disease positive case with an immunochromatographic rapid test to diagnose Leishmaniasis has been reported before [36]. The presence of leishmaniasis in the regions studied herein is very low as only 4 cases were registered during 2017–2018 (communication of SEDES Tarija, Ministry of Health, Bolivia).

In summary, the results obtained in this work would support the combined use of RDTs, in agreement with current recommendation, for delivering a conclusive diagnosis of Chagas disease in the region. We have shown that such tests can be used by mobile teams in the form of screening campaigns relying on a little volume of finger pricked whole blood as sample. Even though further studies are yet required to address the use of RDTs in areas with lower prevalence of *T*. *cruzi* and/or co-circulation of closely related pathogens, the results from this work encourage the use of RDTs as an alternative to conventional serological methods in the Bolivian Chaco and other areas with similar epidemiological characteristics.

## Supporting information

S1 Table Screening campaigns and study sites localizations in and around each of the two cities of Villa Montes and Yacuiba. (XLSX)

S1 File STARD checklist. (DOCX)
